# IRF5 Is a Specific Marker of Inflammatory Macrophages *In Vivo*

**DOI:** 10.1155/2013/245804

**Published:** 2013-12-23

**Authors:** Miriam Weiss, Katrina Blazek, Adam J. Byrne, Dany P. Perocheau, Irina A. Udalova

**Affiliations:** ^1^Kennedy Institute of Rheumatology, University of Oxford, Roosevelt Drive, Headington, Oxford OX3 7FY, UK; ^2^Leukocyte Biology Section, National Heart and Lung Institute, Sir Alexander Fleming Building, Faculty of Medicine, Imperial College, South Kensington, London SW7 2AZ, UK

## Abstract

Macrophages are an integral part of the innate immune system and key players in pathogen clearance and tissue remodelling. Both functions are accomplished by a pivotal network of different macrophage subtypes, including proinflammatory M1 and anti-inflammatory M2 macrophages. Previously, our laboratory identified the transcription factor interferon regulatory factor 5 (IRF5) as the master regulator of the M1 macrophage polarisation. IRF5 was found to be highly expressed in human M1 compared to M2 macrophages. Furthermore, IRF5 dictates the expression of proinflammatory genes such as *IL12b* and *IL23a* whilst repressing anti-inflammatory genes like *IL10*. Here we show that murine bone marrow derived macrophages differentiated *in vitro* with GM-CSF are also characterised by high levels of IRF5 mRNA and protein and express proinflammatory cytokines upon LPS stimulation. These macrophages display characteristic expression of M1-marker MHC II but lack the M2-marker CD206. Significantly, we develop intracellular staining of IRF5- expressing macrophages and utilise it to recapitulate the *in vitro* results in an *in vivo* model of antigen-induced arthritis, emphasising their physiological relevance. Thus, we establish the species-invariant role of IRF5 in controlling the inflammatory macrophage phenotype both *in vitro* and in *in vivo*.

## 1. Introduction

Macrophages are immune cells involved in recognition of pathogenic stimuli and the initiation and resolution of inflammation. They can adapt to various different environmental signals giving rise to several subtypes with distinct functions [1]. These subtypes can be classified as M1 (classically activated) and M2 (alternatively activated) macrophages. In addition, there are several phenotypes associated with M2 macrophages, for example, M2-like or tumour associated macrophages [2]. M1 macrophages secrete high levels of IL-12 and IL-23 but low levels of IL-10, whereas M2 macrophages secrete low levels of IL-12 and IL-23 but high levels of IL-10 [3].

Several reports have described the *in vitro* differentiation of lineage-defined macrophages. In general, these methods utilise M-CSF (macrophage colony stimulating factor; CSF-1) to differentiate bone marrow derived progenitors, followed by priming with various stimuli. Addition of interferon-*γ* followed by lipopolysaccharide (LPS) stimulation has been used to acquire M1 macrophages whereas addition of IL-4 or IL-13 without LPS yields M2 macrophages [3]. Another established method uses GM-CSF (granulocyte/macrophage colony stimulating factor) in order to generate M1 macrophages or alternatively M-CSF treatment for M2 differentiation, usually followed by LPS challenge for both subtypes [4, 5]. In the physiological situation, M-CSF is detected in low steady state levels whereas GM-CSF has been shown to be increased upon stimulation with inflammatory stimuli, such as IL-1, TNF, or LPS [6, 7].

Macrophages are also known to play a key role in autoimmune diseases such as rheumatoid arthritis (RA), a degenerative disease characterised by joint inflammation and bone destruction [8]. At the site of inflammation, macrophages are present in high numbers and it has been found that depletion ameliorates disease severity [9–11]. More specifically, M1 macrophages contribute to RA pathogenesis by secreting proinflammatory cytokines and thereby taking part in the Th1/Th17 response [12, 13].

Distinct macrophage subtypes are not only characterised by their differences in cytokine release but also display differential expression of key transcription factors. Recently, we identified the transcription factor interferon regulatory factor 5 (IRF5) as the major regulator of proinflammatory M1 macrophage polarisation [14]. IRF5 directly induces the expression of proinflammatory cytokines such as IL-6, IL-12b, and IL-23a whilst repressing transcription of anti-inflammatory cytokines such as IL-10 [14, 15]. IRF5 is involved in various inflammatory processes such as the type I interferon response to virus infection and pathogen recognition receptor signalling [16]. Upon viral infection, IRF5 is phosphorylated and thereby translocated to the nucleus where it binds to the regulatory regions of its target genes [17]. Nonviral stimulation of toll-like receptors (TLR) including TLR4, 7, and 9 also leads to activation of IRF5 [16]. Moreover, polymorphisms in the *IRF5* gene have been found to associate with RA [18, 19].

Despite the major role IRF5 plays in macrophage activation, it has rarely been used to track inflammatory macrophages in disease. In this study, we aim to characterise murine macrophages and IRF5 expression in both *in vitro* and *in vivo* models of inflammation. We therefore used the murine model of antigen-induced arthritis (AIA) in which mice are immunised with methylated BSA (mBSA) prior to intra-articular injection of mBSA in one knee, leading to localised inflammation and a Th17 response [20, 21]. First, we analysed *in vitro* differentiated macrophages regarding their IRF5 expression, LPS response, and surface receptor expression. We then used flow cytometry to label intracellular IRF5 in both the *in vitro* macrophages and those derived from the affected knee of the AIA mouse model.

## 2. Material and Methods

### 2.1. Animals and Antigen-Induced Arthritis

For this study wild type mice were bred on a C57Bl/6 background. The experimental animal procedures used in this work were approved by the Kennedy Institute of Rheumatology Ethics Committee and the UK Home Office.

We induced arthritis as described previously; briefly, at day zero, mice were sedated using inhaled isoflurane anaesthesia and subsequently immunised with 100 *μ*g of mBSA emulsified in 0.2 mL of complete Freund's adjuvant, administered intra-dermally at the base of the tail. At day seven, we induced arthritis by means of an intraarticular injection of mBSA (200 *μ*g in 10 *μ*L of sterile PBS), or PBS alone using a sterile 33-gauge microcannula, in sedated animals. At day nine, the mice were sacrificed and the knee joints were excised.

### 2.2. *In Vitro* Differentiation of Macrophages

For the generation of *in vitro* differentiated macrophages, bone marrow from wild type mice was cultured in RPMI-1640 medium with L-glutamine (PAA Laboratories) supplemented with 10% FCS, 1% penicillin/streptomycin, 0.01% 2-mercaptoethanol, and with either recombinant murine GM-CSF (20 ng/mL; Peprotech) or recombinant human M-CSF (100 ng/mL; Peprotech). After eight days, adherent cells were washed with PBS and replated, then stimulated with LPS (100 ng/mL; Alexis Biochemicals).

### 2.3. RNA Extraction and Quantitative Real-Time PCR

Total RNA was extracted using RNeasy Mini Kit (Qiagen) as per the manufacturer's instructions. Contaminating genomic DNA was removed from RNA samples using the RNase-Free DNase Set (Qiagen). Total RNA was reverse-transcribed into cDNA using the High Capacity cDNA Reverse Transcription Kit (Life Technologies) as per the manufacturer's instructions. Real-time PCR reactions were performed on an ABI 7900HT (Life Technologies) with TaqMan primer sets for murine *Fizz1*, *iNOS*, *Il10*, *Il12b*, *Il23a*, *Irf5*, and *Hprt* (Life Technologies) and gene expression was analysed using the change-in-threshold ΔΔCt-method.

### 2.4. Western Blot

For protein isolation, cells were harvested with Versene (EDTA) 0.02% (Lonza). Pellets were resuspended with macrophage lysis buffer (20 mM Tris pH 8, 300 mM NaCl, 1% NP40, and 10% glycerol) containing freshly added protease inhibitors (Roche). Samples were incubated on ice for 30 min before cellular debris was removed by centrifugation for 15 min, at 13,000 rpm/4°C. Lysates were transferred into new tubes and stored at −80°C. To determine the protein concentration of whole cell lysates a BCA test (Thermo Scientific) was performed according to the manufacturer's instructions.

5–7 *μ*g of total protein were resolved by Novex Tris-glycine gel (Life Technologies), transferred onto a PVDF membrane (GE Healthcare) by wet western blotting, and subjected to incubation with rabbit anti-IRF5 (Abcam) or mouse anti *β*-actin (Sigma), followed by detection with horseradish-peroxidase- (HRP-) conjugated secondary antibodies and chemiluminescent substrate solution ECL (GE Healthcare).

### 2.5. Enzyme Linked Immunosorbent Assay (ELISA)

Supernatants of stimulated cells were transferred into tubes, centrifuged for 5 min at 3,300 rpm, and stored at −20°C until needed. Cytokine secretion was quantified for murine IL-10 (eBioscience), IL-12p70 (eBioscience), and IL-23 (eBioscience) according to the manufacturer's instructions. Absorbance was read at 450 nm by a spectrophotometric ELISA plate reader (Labsystems Multiscan Biochromic) and analysed using Ascent Labsystems software. All samples were analysed in triplicate in a volume of 50 *μ*L.

### 2.6. Flow Cytometry

Single cell suspensions of *in vitro* differentiated macrophages and knees were washed with FACS buffer (1% BSA, 0.01% sodium azide in PBS, and pH 7.4) and stained with the following antibodies: APC conjugated anti-CD206 antibody (BioLegend), APC-Cy7 conjugated anti-CD11b antibody (BD Biosciences), PE conjugated anti-MCH II [I-A/I-E] antibody (BD Biosciences), PerCP conjugated anti-CD45 antibody (BD Biosciences), and PE-Cy7 conjugated anti-F4/80 (eBioscience). For intracellular FACS staining, cells were fixed with fixation/permeabilisation solution (eBioscience) and washed with permeabilisation buffer (eBioscience). Samples were then stained with rabbit anti-IRF5 antibody (Abcam) followed by secondary staining with goat anti-rabbit Alexa Fluor 488 (Life Technologies). FACS analysis was performed using a FACS Canto II (BD Biosciences), and the data were analysed with Flow Jo software, version 7.6 (Treestar).

### 2.7. Statistical Analyses

Statistical analysis was performed using GraphPad v5.0 (GraphPad Software) using two-way ANOVA (with Bonferroni's multiple comparisons) or unpaired one-tailed Mann-Whitney *U* tests (comparisons between two groups). *P* values less than 0.05 were considered significant.

## 3. Results and Discussion

### 3.1. High Levels of IRF5 Expression in Murine GM-CSF Differentiated Bone Marrow Derived Macrophages

In order to assess the expression of IRF5 *in vitro*, bone marrow derived macrophages were differentiated with either GM-CSF or M-CSF (GM-BMDM and M-BMDM, resp.). After nine days of differentiation, macrophages were challenged with LPS for 0 h, 1 h, 4 h, 8 h, and 24 h and analysed for mRNA and protein levels of IRF5.

**Figure 1:**
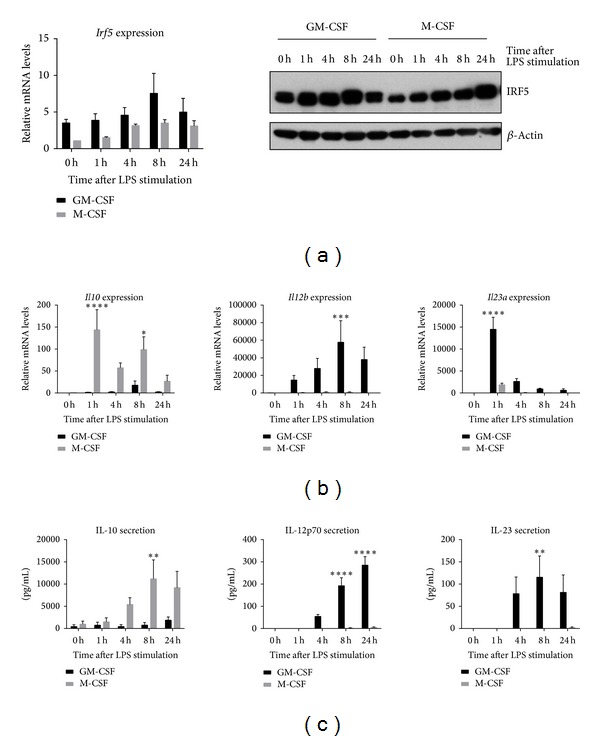
IRF5 levels and cytokine response of *in vitro* differentiated macrophages. BMDMs were differentiated with GM-CSF (20 ng/mL) or M-CSF (100 ng/mL) for eight days. All cells were challenged with LPS for the indicated time periods. (a) Transcript levels were measured with real-time PCR. Error bars represent the standard error for *n* = 6. Protein levels of IRF5 and *β*-actin were determined by western blot. Experiment is representative for three independent experiments. (b) and (c) At each time point RNA (top panel) and supernatants (bottom panel) were collected. Error bars represent the standard error for *n* = 5. Statistical analysis was performed by 2-way ANOVA and Bonferroni's multiple comparison. *P ≤ 0.05; **P ≤ 0.01; ***P ≤ 0.001; ****P ≤ 0.0001.

In unstimulated murine cells, IRF5 levels were considerably higher in GM-CSF differentiated compared to M-CSF differentiated macrophages (Figure 1(a)). Interestingly, this expression pattern is also exhibited by their unstimulated human macrophage counterparts, with significantly higher IRF5 expression in GM-CSF *in vitro* differentiated human macrophages compared to those differentiated with M-CSF [14].

Upon LPS stimulation, IRF5 mRNA and protein expression were induced in M-CSF differentiated murine cells and further induced in GM-CSF differentiated murine cells. *Irf5* mRNA levels increased between 4 and 8 h but protein levels were already higher after 1 h of poststimulation (Figure 1(a)) we therefore hypothesised that the LPS-induced production of IRF5 was most likely due to a combination of two factors: (1) increased mRNA levels and (2) protein stabilisation, possibly related to activation by phosphorylation or ubiquitination [22, 23]. IRF5 has been shown to be essential for the proinflammatory phenotype of human monocyte derived GM-CSF macrophages upon LPS stimulation. However, mRNA and protein levels in human M-CSF derived macrophages are not further induced upon LPS stimulation, suggesting some species-specific or cell source-specific differences in LPS-regulated IRF5 production.

### 3.2. Distinct Cytokine Expression Profiles of M-CSF and GM-CSF Derived BMDMs

Next, to determine the inflammatory properties of *in vitro* differentiated murine macrophages, expression and secretion of the cytokines IL-10, IL-12, and IL-23 were analysed.

As expected, each macrophage subtype was found to display differential behaviour to LPS stimulation regarding their cytokine expression (Figures 1(b) and 1(c)). Transcription and secretion of the anti-inflammatory cytokine IL-10 were elevated in M-CSF differentiated macrophages compared to GM-CSF treated cells. LPS stimulation of M-BMDMs resulted in increased IL-10 expression on both transcript and protein level. At 24 h, *Il10* mRNA returned to an almost basal level, whereas protein secretion remained high. IL-10 protein secretion was significantly higher following 8 h LPS stimulation in M-BMDMs whereas GM-BMDMs only showed basal IL-10 expression.

Proinflammatory cytokines IL-12 and IL-23 were found to be expressed at much higher levels in GM-CSF derived macrophages whereas M-BMDMs show only minimal expression of proinflammatory cytokines, although with similar kinetics of expression as in GM-BMDMs (Figure S1A in Supplementary Material available online at http://dx.doi.org/10.1155/2013/245804). The differences in cytokine expression were statistically significant on both the transcript and protein levels. *Il12b* mRNA was induced upon LPS stimulation in GM-BMDMs, with the highest levels observed 8 h after stimulation. Secretion of IL12p70 was increased from 4 h of stimulation onwards. Although M-BMDMs expressed low levels of *Il23a* mRNA following 1 h of stimulation, they did not secrete heterodimeric IL-23 protein at any time point. In GM-BMDMs *Il23a* mRNA expression peaked following 1 h of LPS stimulation, while IL-23 protein secretion extended to 24 h after LPS stimulation.

We also noted that IRF5 levels increased in M-BMDMs upon LPS stimulation but did not result in significant induction of proinflammatory cytokines. Thus, we hypothesised that this could be due to a lower functional activity of IRF5 in M-BMDMs, as IRF5 protein is subject to posttranslational modifications such as phosphorylation and ubiquitination [22–24]. However, the status of posttranslational modifications for IRF5 in LPS stimulated macrophages is yet to be determined. Furthermore, the availability of activating cofactors potentially required for IRF5 mediated induction of proinflammatory cytokines might be different in M-BMDMs compared to GM-BMDMs.

Thus, consistent with its proposed role as a master regulator of the M1 macrophage phenotype and in accordance with data for human *in vitro* differentiated macrophages [14], GM-CSF differentiated BMDMs express high levels of IRF5 and produce IL-12 as well as IL-23 following stimulation with LPS, whereas M-CSF differentiated BMDMs express lower levels of IRF5 and produce IL-10. These data confirm the study of Fleetwood et al. [4] that suggested that GM-CSF and M-CSF induce a distinct M1 or M2 BMDM phenotype, respectively.

### 3.3. Specific Intracellular IRF5 Staining of M-CSF and GM-CSF Derived BMDMs

In order to establish intracellular IRF5 staining, expression was measured by fluorescence activated cell sorting (FACS) of unstimulated and LPS stimulated GM- and M-BMDMs at day nine of differentiation. Known cell surface receptor markers of M1 and M2 macrophages, MHCII, and CD206 (mannose receptor), respectively, as well as the pan macrophage marker F4/80 were used as controls for specificity of IRF5 staining.

**Figure 2:**
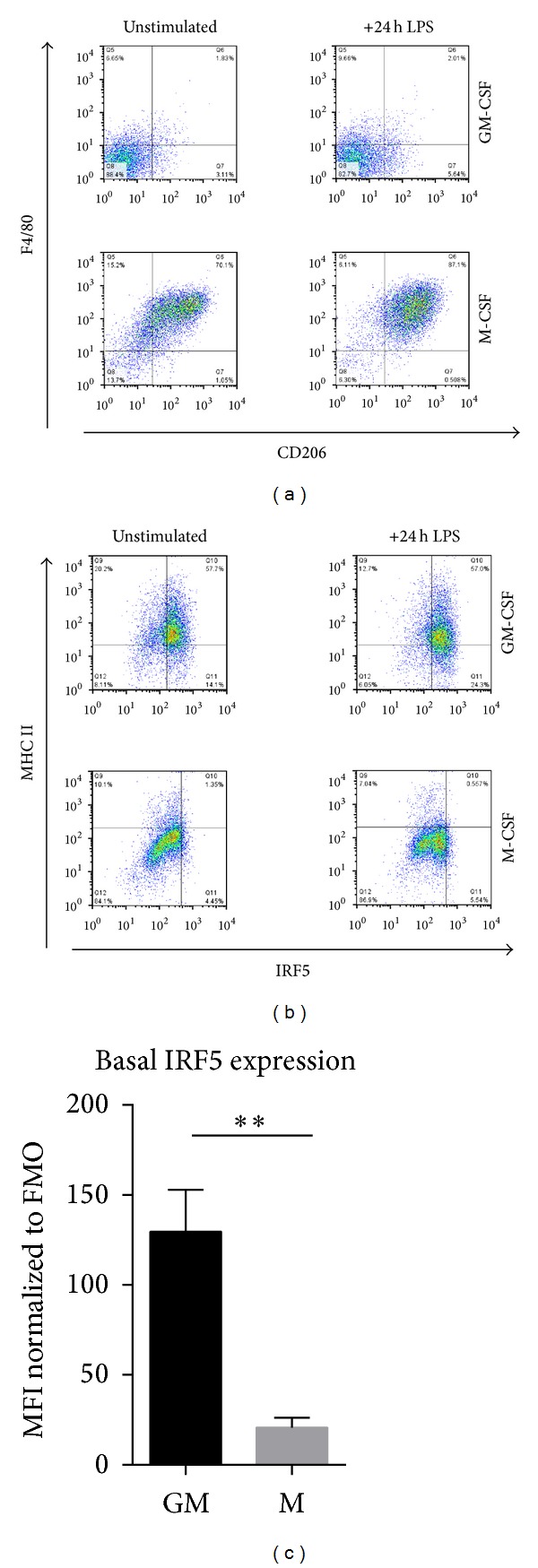
Surface receptor expression of polarised macrophages and intracellular IRF5 staining. Macrophages were *in vitro* differentiated with GM-CSF or M-CSF for eight days and then stimulated with LPS for 24 h. FACS samples were collected before and after stimulation. (a) and (b) Samples were stained for the expression of F4/80, CD206, MHC II, and IRF5. (c) Macrophages were stained for intracellular IRF5 and staining in unstimulated cells was quantified by mean fluorescence intensity (MFI). Error bars represent the standard error for *n* = 6. Statistical analysis was performed by one-tailed Mann-Whitney *U* test. **P ≤ 0.01.

Around 70% of the M-CSF derived macrophages were F4/80^high^ and CD206^high^ (Figure 2(a)). GM-CSF differentiated macrophages on the other hand were generally F4/80^low^ and only 1-2% of them expressed CD206. Although F4/80 is reported to be highly expressed on all tissue macrophages, GM-CSF derived cells only showed a low percentage of F4/80+ cells. This could be because GM-CSF can also induce differentiation into DCs, effectively leading to generation of DC-like macrophages [25]. Conversely, 80% of unstimulated GM-CSF derived BMDMs expressed MHC II, whereas in CD206 positive M2 macrophages only 10% of cells exhibit expression of this marker (Figure 2(b)). A similar distribution was observed for IRF5, where over 70% of unstimulated GM-BMDMs were IRF5+ compared to only 5% of unstimulated M-BMDMs. In summary, most unstimulated M-BMDMs display the M2 marker CD206 and F4/80 whereas GM-BMDMs lack the latter but express M1 markers MHC II and IRF5. Basal IRF5 levels in unstimulated cells were quantified using mean fluorescence intensity (MFI) (Figure 2(c)). The MFI for IRF5 in GM-CSF derived macrophages was found to be sixfold higher than in M-CSF differentiated macrophages. The quantified differences in the IRF5 levels were further confirmed by the analysis of IRF5 mRNA and protein levels in these samples (Figure S1B).

LPS stimulation only minimally increased expression of F4/80 and CD206 in GM-BMDMs, whilst in M-BMDMs the percentage of F4/80^high^  CD206^high^ cells increased to almost 90%. MHC II expression decreased after 24 h of LPS stimulation in both cell types, consistent with the previous reports indicating that LPS does not induce expression of MHC II in macrophages [26–28]. Of significance, the population of IRF5^+^ cells increased to over 80% in LPS-stimulated GM-BMDMs but remained unchanged in M-BMDMs contrary to the observed increase in IRF5 protein levels detected by Western Blot analysis (Figure 1(a)). Although the same antibody is used for both techniques, in a Western Blot, proteins are denatured, whereas in FACS proteins are in a native configuration. It is possible that in M-BMDMs native IRF5 protein is in a conformation that does not allow its recognition by this antibody unless denatured. The structure of proteins can be affected by posttranslational modifications such as phosphorylation or ubiquitination which also dictate protein activity. As highlighted above, the manner in which IRF5 is modified in stimulated macrophages is the subject of ongoing research.

Thus, we have developed intracellular IRF5 staining and demonstrated that M1 macrophages have a higher percentage of IRF5+ cells than M2 macrophages. It is worth noting though that FACS staining for IRF5 in macrophages is challenging due to relatively high background from secondary antibodies and macrophage autofluorescence. A reporter IRF5 mouse strain, similar to the described RelA-GFP knock-in [29], would further facilitate analysis of IRF5 expression in macrophage populations and possibly other cell types in *in vivo* models. In addition, it would be helpful in the analysis of the intracellular localisation of IRF5 in response to stimulation.

### 3.4. IRF5 Expressing Macrophages at the Site of Inflammation in an Experimental Model of Arthritis

**Figure 3:**
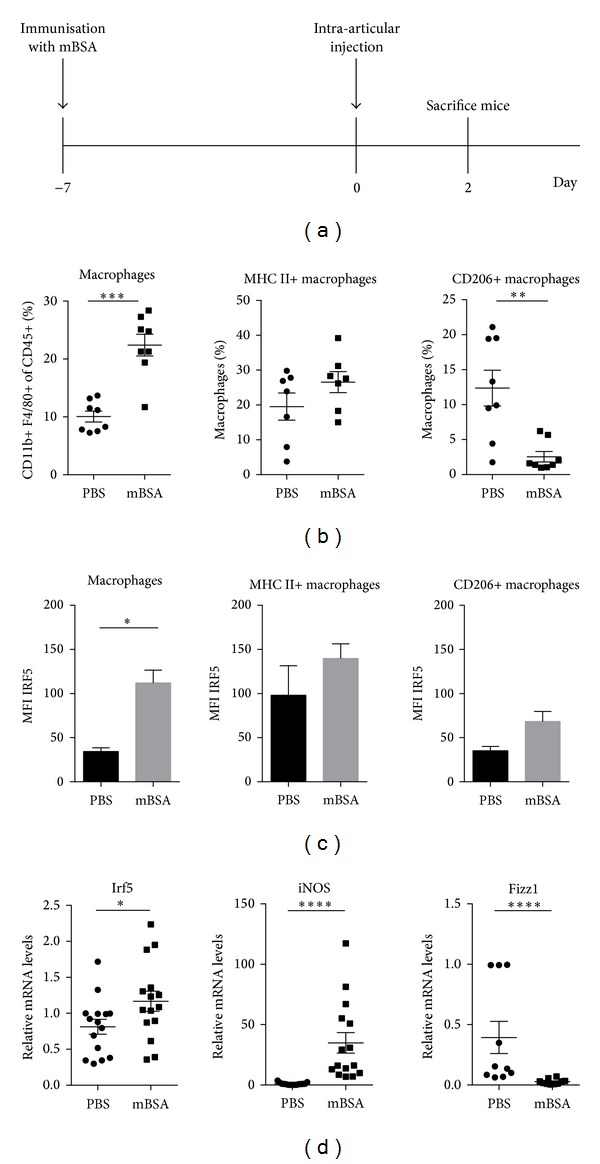
Macrophage populations and IRF5 expression at the site of inflammation in a mouse model of arthritis. Mice were immunised with mBSA in complete Freud's adjuvant prior to intra-articular injection of mBSA or PBS. Knees were collected at day two of disease. (a) Schematic of the experimental set-up for antigen-induced arthritis. (b) Samples from three independent experiments were stained for flow cytometry with antibodies against CD45, CD11b, F4/80, CD206, and MHC II. (c) IRF5 FACS staining was quantified calculating the mean fluorescence intensity in knees of three wild type mice. (d) Total RNA was isolated from knees of three independent experiments and analysed by real-time PCR for expression of Irf5, iNOS, and Fizz1. Statistical analysis was performed throughout by one-tailed Mann-Whitney *U* test. *P ≤ 0.05; **P ≤ 0.01; ***P ≤ 0.001; ****P ≤ 0.0001.

Finally, we utilised a murine model of antigen-induced arthritis to explore the possibility of using IRF5 as a marker of inflammatory macrophages in a disease setting. Mice were immunised with mBSA and after seven days arthritis was induced by intra-articular injection of mBSA (affected knee) or PBS (control knee) (Figure 3(a)). Affected knees and control knees were harvested two days after injection and subjected to FACS analysis. In addition, RNA was isolated from knees to study mRNA levels of *Irf5* and macrophage markers at the site of inflammation. The chosen markers were *iNOS* and *Fizz1* for M1 and M2 macrophages, respectively [30, 31].

Macrophages were defined as CD45+, CD11b+, and F4/80+ cells. Within this population, we identified proinflammatory (MHC II+ CD206−) and anti-inflammatory (MHC II-CD206+) macrophage subsets. The percentage of total macrophage populations, as well as the proinflammatory macrophage subset, was significantly increased in inflamed knees compared to control knees (Figure 3(b)). In contrast, the percentage of CD206+ macrophages was found to be significantly reduced after antigen challenge. These results also demonstrate that there are a large number of macrophages which do not fit either category. This probably reflects the extent of macrophage plasticity and the wide spectrum of *in vivo* macrophage subtypes [32]. This especially holds true in a disease setting where incoming macrophages might be at different stages of polarisation and where the inflammatory environment can be constantly changing.

Quantification of IRF5 FACS staining in macrophages demonstrated that increased IRF5 expression can be detected in affected knees (Figure 3(c)). When IRF5 levels were assessed in each macrophage population individually, it was observed that proinflammatory macrophages express relatively high levels of IRF5. CD206+ macrophages express less IRF5 but also show a minor increase in inflamed knees, suggesting that the remaining CD206+ macrophages at the site of inflammation express more IRF5 than they did prior to challenge. The *in vivo* data confirm the findings in *in vitro* differentiated macrophages that proinflammatory macrophages do express higher levels of IRF5 than CD206+ macrophages.

Analysis of whole knee RNA extracts supported these observations and demonstrated that *Irf5* transcript levels are significantly augmented in affected knees (Figure 3(d)). Expression of the M1 marker *iNOS* was significantly higher in mBSA injected knees whereas *Fizz1* expression is diminished. Taken together, these results indicate that there is an increasing amount of proinflammatory macrophages at the site of inflammation which correlates with an increase in IRF5 mRNA and protein. We therefore conclude that IRF5 is an appropriate marker for detection of inflammatory macrophages in this arthritis disease model. However, it has to be kept in mind that although IRF5 levels within macrophage populations increase, this may not necessarily translate into elevated protein activity since the phosphorylation status and cellular localisation are not taken into account. It has been shown that IRF5 undergoes posttranslational modifications and is regulated by phosphorylation and ubiquitination [22–24]. However, the role of IRF5 activation in the context of disease has not been studied extensively and further research will be required to elucidate this [33].

Recently, IRF5 was used as an indicator for M1 macrophage infiltrate in house dust mite induced asthma animal models [34]. Although this study did not describe the phenotype of the IRF5 expressing macrophages in detail, it demonstrated that IRF5 can potentially be used as a marker in a different disease setting and tissue. This is particularly important since IRF5 associates not only with RA but also with several other autoimmune diseases such as inflammatory bowel disease, asthma, and systemic lupus erythematosus [35–38].

It has recently become clear that in addition to macrophages derived from infiltrating monocytes generated in bone marrow, tissue-resident macrophages of different origin may also play a crucial role in inflammation [39, 40]. Moreover, transcriptional profiling of macrophages from different origins demonstrated heterogeneity of macrophage populations and revealed tissue-specific transcriptional signatures [32]. This suggests that identification of subset specific transcription factors is needed to tease out the contribution of different macrophage subtypes in inflammatory processes, especially in disease-related chronic inflammation or autoimmunity that so far received less attention [41]. We hypothesise that IRF5 could play a critical role in tracking inflammatory macrophages in various inflammatory diseases.

## 4. Conclusions

To conclude, this study clearly demonstrates that IRF5 is highly expressed in murine proinflammatory macrophages and may be utilised as a reliable marker for macrophages at sites of inflammation. Murine GM-BMDMs express IRF5 and proinflammatory cytokines *in vitro* when challenged with LPS. We show that it is possible to label intracellular IRF5 in these proinflammatory macrophages, as well as in macrophages in an inflamed knee during the progression of an experimental mouse model of antigen-induced arthritis. Thus, this study describes a useful method for tracking proinflammatory macrophages and demonstrates its feasibility in a murine disease model.

## Supplementary Material

Pro-inflammatory cytokine expression in M-BMDMs and IRF5 expression in samples used for intracellular IRF5 staining. A. M-CSF differentiated cells were challenged with LPS for the indicated time periods. At each time point RNA (top panel) and supernatants (bottom panel) were collected. Error bars represent the standard error for *n*=5. These data are the same as in Figure 1A but exclude the GM-CSF data set. B. Irf5 transcript levels were measured by Real-time PCR. Error bars represent the standard deviation of experimental duplicates. Protein levels of IRF5 and **β**-actin were determined by western blot. RNA and protein samples were from an experiment used to generate data for Figure 2C.
