# Supplementary material for: IRF5 Is a Specific Marker of Inflammatory Macrophages In Vivo
Source: Mediators Inflamm. 2013 Dec 23;2013:245804. doi: 10.1155/2013/245804 (PMC3885211; doi:10.1155/2013/245804)
Supplement: Supplementary file 1 — Pro-inflammatory cytokine expression in M-BMDMs and IRF5 expression in samples used for intracellular IRF5 staining. A. M-CSF differentiated cells were challenged with LPS for the indicated time periods. At each time point RNA (top panel) and supernatants (bottom panel) were collected. Error bars represent the standard error for n=5. These data are the same as in Figure 1A but exclude the GM-CSF data set. B. Irf5 transcript levels were measured by Real-time PCR. Error bars represent the standard deviation of experimental duplicates. Protein levels of IRF5 and β-actin were determined by western blot. RNA and protein samples were from an experiment used to generate data for Figure 2C. [file 245804.f1.pdf]

Figure S1

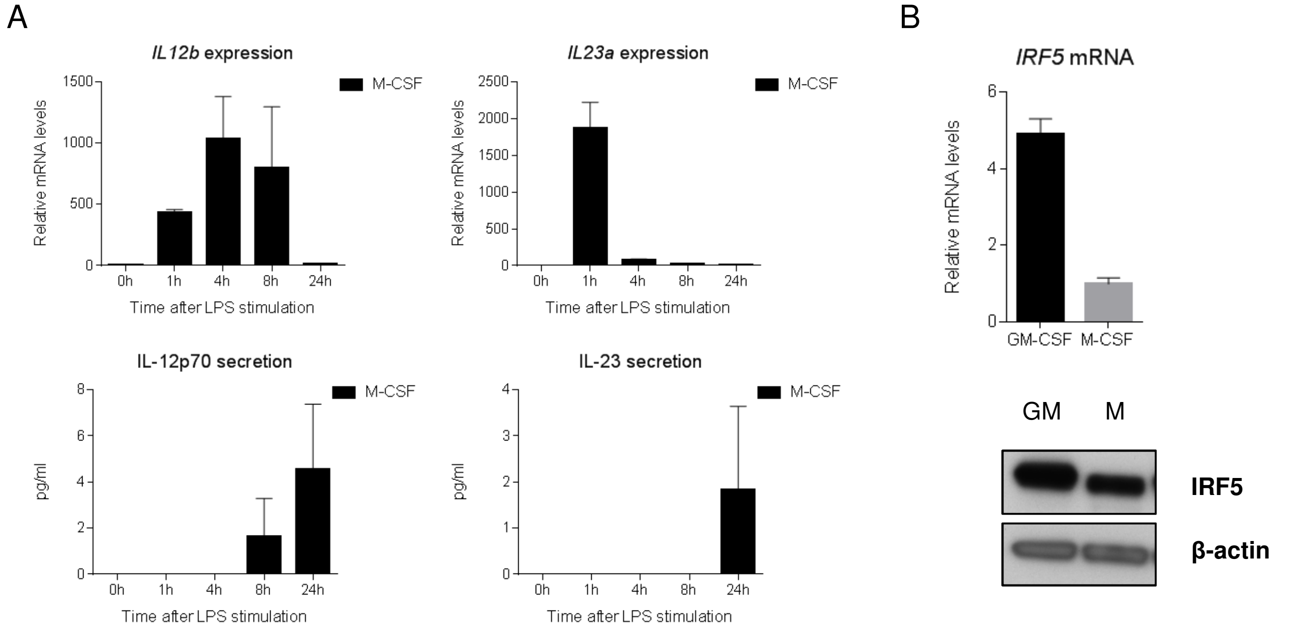

FIGURE S1: Pro-inflammatory cytokine expression in M-BMDMs and IRF5 expression in samples used for intracellular IRF5 staining.

BMDMs were differentiated with GM-CSF (20ng/ml) or M-CSF (100ng/ml) for eight days. **A.** M-CSF differentiated cells were challenged with LPS for the indicated time periods. At each time point RNA (top panel) and supernatants (bottom panel) were collected. Error bars represent the standard error for n=5. This data is the same as in Figure 1A but excluding the GM-CSF data set. **B.** *Irf5* transcript levels were measured by Real-time PCR. Error bars represent the standard deviation of experimental duplicates. Protein levels of IRF5 and β-actin were determined by western blot. RNA and proten samples were from an experiment used to generate data for Fig 2C.
